# The Use of Blockchain Technology in Public Health: Lessons Learned

**DOI:** 10.7759/cureus.63198

**Published:** 2024-06-26

**Authors:** Hemlata Sahu, Sonali Choudhari, Swarupa Chakole

**Affiliations:** 1 Department of Community Medicine, School of Epidemiology and Public Health, Jawaharlal Nehru Medical College, Datta Meghe Institute of Higher Education and Research, Wardha, IND

**Keywords:** decentralized, health insurance, electronic health records, telemedicine, digital public health

## Abstract

Blockchain is a new technology utilized to develop creative solutions in different industries, such as health care. Blockchain is a decentralized and distributed encrypted system made up of interconnected blocks containing transaction-related information that can be shared with network participants. A blockchain network is utilized in the healthcare industry to safeguard and share patient information among hospitals, pharmacies, and doctors' diagnostic labs. Blockchain applications can precisely identify serious and potentially harmful mistakes within the medical sector. The objective is to comprehensively explore the potential use, present implementations, challenges, and future possibilities of blockchain in health management systems, and to provide information to researchers, policymakers, and practitioners on how to utilize new technology to enhance data security, efficiency, decentralization of data, authenticity of data, transparency, and verifiability of data compared to conventional databases in health management systems. Key review findings for blockchain technology in public health surveillance might include enhanced data security and accessibility of data, data storage and sharing, ensuring tamper-proof records are accessed, empowering patients, and improving overall healthcare outcomes. Its immutability proves to be important for securing healthcare data. It offers a safeguard for health records and clinical trial outcomes and ensures compliance with regulatory standards. This evaluation focuses on how it has transformed data protection, improved workflows, and safe health information interchange. Despite obstacles, further study and standardization initiatives have the potential to transform health care and guarantee patient care that is resilient and trustworthy. In the present healthcare industry, blockchain technology plays an essential role in healthcare systems. It can lead to computerized processes for collecting and validating data, accurate information collected from multiple sources, and data that are fixed, transparent to misuse, and secure, with a reduced risk of digital crimes. In addition, the study provides a detailed analysis of the potential applications for including the use of blockchain technology in transforming public health surveillance.

## Introduction and background

A blockchain network is utilized in the healthcare industry to safeguard and transfer patient information among hospitals, diagnostic laboratories, pharmacy companies, and doctors [1]. Thus, blockchain is a developing technology utilized for generating new solutions across different industries, such as health care. It is a decentralized digital ledger system where the verification process is distributed through a network of independent entities to ensure data security, transparency, and efficiency. Such decentralized technology offers a possible solution for several health-related issues, starting from safe data transfer to computerized verification procedures. New application areas are developed for blockchain technology, including authenticity, confidentiality, and transparency, bringing cryptography to enhance both the security and verifiability of data to levels higher than the ones provided by traditional databases. However, the proliferation of blockchain technology adoption within health care is at a very nascent stage, and very few research and discussion articles have been penned down to date [2].

Blockchain technology has emerged as an innovative creation with the power to revolutionize data management and enhance security across various industries [3]. More specifically, blockchain could significantly impact medical imaging research and clinical practices. This includes independent validation and verification, utilizing research and machine learning methods, implementing smart contracts, and enabling the transfer of medical data and images across different domains among patients, doctors, and institutions [4]. Enhancing security, privacy, and confidentiality for medical data is one of the major benefits of implementing blockchain technology in health care. This becomes especially important in light of the general lack of strong safeguards inside healthcare institutions to prevent unauthorized access to patient data [5]. Furthermore, the current electronic healthcare record (EHR) system may fall short of meeting the necessary standards to protect patient privacy. Blockchain is seen as a workable answer to these problems, providing advantages over systems that depend on password-based authentication and security. Better control over information, including patient identity, within computer networks can be achieved by improving identity and access management [6].

The challenges in delivering health care have become a worldwide issue, necessitating proportionate solutions to improve the quality of healthcare services [1]. The era of big data is revolutionizing health care by shifting from a reactive model, where individuals seek treatment for existing issues, to a proactive one. Through real-time monitoring of diverse health data, early predictions and insights can be derived, marking a departure from the conventional practice of seeking health care only when problems surface. This transformation aims to foster a more preventative and comprehensive approach to healthcare delivery [7]. The conventional healthcare system encounters significant hurdles in efficiently handling patient data, with concerns ranging from data security and interoperability to maintaining patient privacy. These challenges are especially relevant in dentistry, given the intricate data ecosystems formed by the integration of diverse systems like EHR, imaging systems, and insurance networks [8]. The age of big data can revolutionize healthcare delivery, shifting from a reactive model that addresses issues when they arise to a proactive approach. This involves real-time monitoring of health data for predictions and insights, promoting regular checkups, and continuous health management [7]. The adoption of these technologies in communities has the potential to transform public health surveillance and finally provide a window into the socioeconomic factors that influence health, enabling the prevention of disease outbreaks and other circumstances, such as pandemics, for public health agencies. These qualities make blockchain a unique technology for the outside observer - a decentralized facet and accessibility solution rather than a problem for the healthcare sector. Blockchain covers far more than the indication of information sources from multiple pertinent elements and attaches them to aspects of the healthcare field, embracing entire categories such as wearables [9].

Blockchain technology transforms prescriptions by providing visibility from the drug’s fabrication to the pharmacy shelves. With the blockchain network assembled, congestion and freight direction paths and speeds can be monitored carefully. Such data lets hospitals more accurately forecast the timeframes they need to make purchases, preventing last-minute purchases and shortfalls. Blockchains based on digital networks ensure the incorruptibility of logistic records, which promotes confidence as it eliminates fraud between data management and transactions through record payments and pharmaceuticals. In the end, it reduces patient spending without compromising performance and eliminates the need for multi-level authentication blockers [10-12].

The key attribute of blockchain, its immutability, proves to be crucial for securing healthcare data. It offers a safeguard for health records and clinical trial outcomes and ensures adherence to regulatory standards. The utilization of smart contracts illustrates how blockchain can be employed to facilitate real-time patient monitoring and interventions in the medical field [13]. Blockchain technology is one potential solution to address or lessen these issues [14]. Using this technique, timestamped transactions can be recorded in a distributed virtual ledger. Cryptography is employed to secure blockchain transactions, preventing tampering. This makes the blockchain a tamper-resistant digital ledger, providing all participants with an unchangeable version of the truth. It is particularly effective for tracking assets and establishing trust, as seen in scenarios like managing health data or obtaining user consent for data collection [15]. Blockchain technology in public health surveillance has demonstrated encouraging outcomes and important teachings. Blockchain guarantees the authenticity of medical information, enhances cooperation among healthcare professionals and organizations, and allows for immediate tracking of trends in public health. It also improves the safeguarding of privacy and the tracking of supply chain movements.

As a result, blockchain technology uses cryptography to ensure data security, authenticity, public health monitoring, transparency, and verifiability when compared to traditional databases. Advancements in this field have produced creative use-case applications across a range of industries. However, despite its rapid growth, the use of blockchain technology in health care in general is still in its early stages and has received little research and discussion [2].

## Review

Methodology

This narrative review explores the role of blockchain in public health, i.e., in enhancing and securing healthcare data, streamlining processes, and empowering patients in the delivery of quality health care, surveillance, public health emergencies, etc. Our search encompassed databases such as PubMed (Medline) and Google Scholar, along with government websites like the Ministry of Health and Family Welfare, the Ministry of Commerce and Industry, the Ministry of Electronics and Information Technology, the Ayushman Bharat Digital Mission, and the National Health Portal of India.

The PubMed search strategy tailored was as follows: ("public health"[Title/Abstract] OR "Public health technology"[Title/Abstract] OR "Public Health Surveillance"[Title/Abstract] OR "public health management"[Title/Abstract] OR "Public health emergency"[Title/Abstract] OR "Electronic health records"[Title/Abstract] OR "health informati*"[Title/Abstract] OR "Healthcare System"[Title/Abstract] OR "public health"[MeSH Terms]) AND ("Blockchain"[Title/Abstract] OR "Blockchain"[MeSH Terms]).

Identifying pertinent articles involved full-text searches in the last 10 years. We also examined cross-references in the bibliography of the searched literature. The review encompasses studies published between 2012 and 2023, with the removal of duplicates, abstracts, works written in non-English, unpublished material, and references that did not align with the objective and scope of the topic under review.

Blockchain in health care

Blockchain in health care spans electronic health records, pharmaceutical distribution networks, medical studies, monitoring patients from a distance, health insurance reimbursements, analyzing data, and other possible uses within the industry. The healthcare issues concerning EHR, supply chains, health insurance, genomics, and consent management are connected. As an example, utilizing blockchain technology to tackle problems in genomics and EHR presents comparable obstacles relating to data ownership, sharing, and setting up a platform for data exchange between owners and purchasers. Consent management and EHR can work together, with blockchain-based consent solutions as access control. This could potentially allow researchers and healthcare providers to access individual health records stored in off-chain databases through blockchain infrastructure, contingent on obtaining consent from the individual [15]. We illustrate the significant contribution of blockchain during the COVID-19 pandemic, offering insights that can guide the prevention of future major infectious diseases. We explore the incorporation and utilization of emerging technologies and blockchains, aiming to outline a connected healthcare ecosystem.

Application, Advantages, and Uses of Blockchain in Health Care

**Figure 1:**
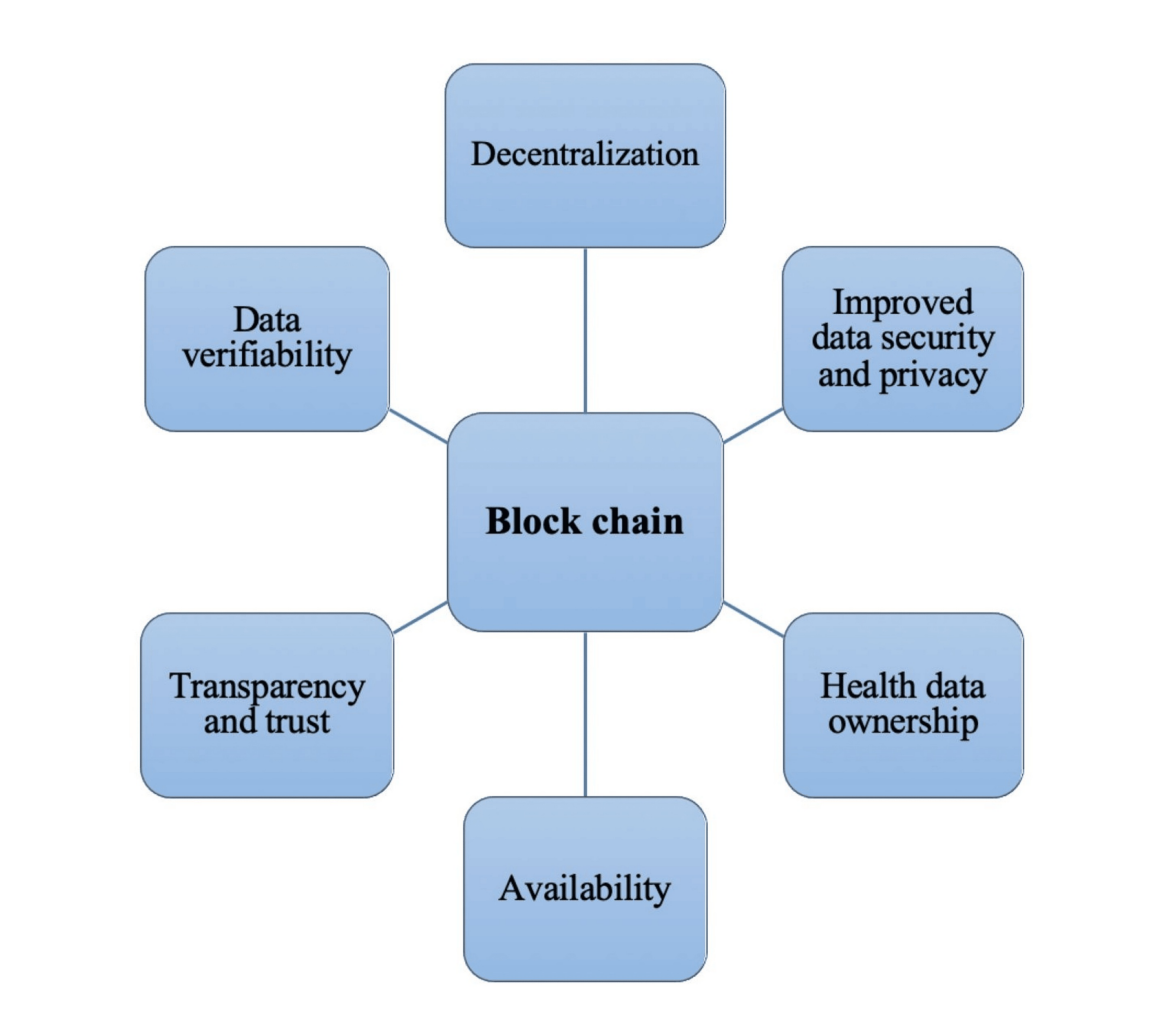
Applications of blockchain in health care. Image credit: Hemlata Sahu.

According to the results of this narrative review, most research in healthcare environments on blockchain technology is primarily centered around managing electronic health records. Following this, notable attention was given to biomedical research and education, remote patient monitoring, pharmaceutical supply chains, health insurance claims, health data analytics, and other potential applications. Figure 1 shows the applications of blockchain in health care.

**Figure 2:**
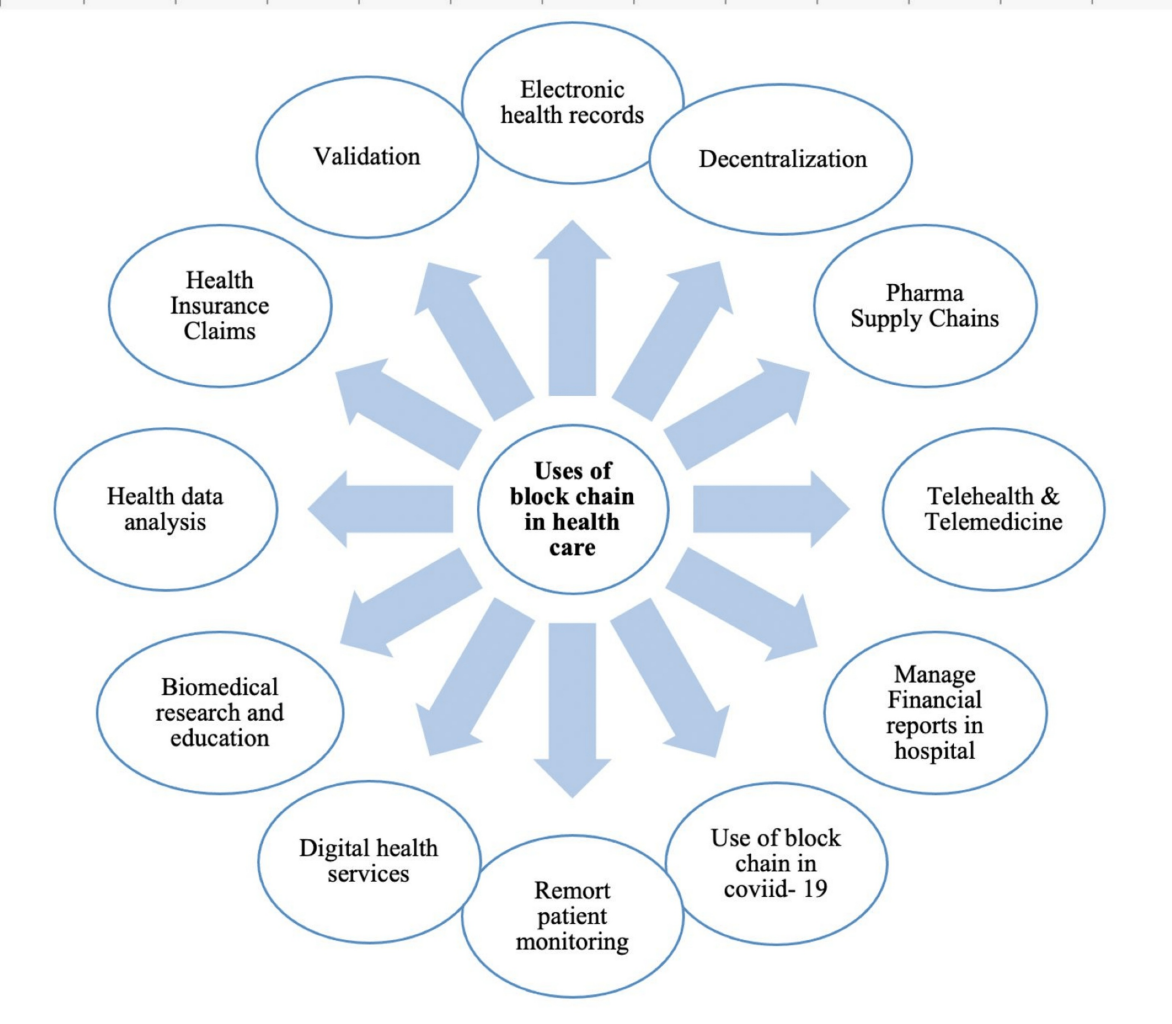
Uses of blockchain in health care. Image credit: Hemlata Sahu.

Figure 2 shows the uses of blockchain in health care. The use of blockchain technology in public health surveillance holds great promise for improving data management, transparency, and response capabilities, it is essential to carefully evaluate its practical implications and address potential challenges to realize its full potential in transforming public health surveillance systems.

Electronic Health Records

Electronic health records involve electronically modeling, storing, and managing individuals' health-related data. Traditionally, these records are stored separately by service providers, giving them control and potentially limiting data sharing with other healthcare stakeholders [16]. The transparent nature of blockchain creates auditable records of public health surveillance activities, enhancing accountability and trust among stakeholders.

Blockchain technology in EHR management facilitates seamless, transparent, and trustworthy sharing of data among healthcare stakeholders, empowering patients with control over their information. The features of blockchain, including decentralization, immutability, data provenance, reliability, robustness, smart contracts, security, and privacy, make it suitable for storing and managing patient EHR [17].

The second application that involves the integration of EHR, known as privacy-preserving data sharing utilizing blockchain technology, was introduced by Liu et al. [18]. Implemented on the Ethereum blockchain platform, this system minimizes the likelihood of medical record leakage and enhances the security of data sharing within the healthcare domain.

Biomedical Research, Education, and Clinical Research

Blockchain technology is extensively utilized in the field of biomedical research and education to maintain the confidentiality, authenticity, sharing, and documentation of data, especially in the realm of clinical trials [19]. Each data point or consent incorporated into the blockchain technology is assigned a timestamp and is publicly visible, thanks to cryptographic validation. This is accomplished to ensure transparency. Blockchain allows for the storage of all plans, consents, protocols, and potential outcomes even before the initiation of clinical trials [20].

Validation

In Blockchain, transactions are confirmed through algorithms and added to the chain, ensuring authenticity through encryption and digital signatures. Healthcare sectors are exploring ways to leverage blockchain to enhance safety and reduce costs. Validating results effectively could pave the way for significant advancements in healthcare management through blockchain [21-23].

Remote Patient Monitoring

Another application of blockchain in healthcare involves remote patient monitoring. Typically, remote patient monitoring entails collecting biomedical records from the body and mobile devices, enabling the remote tracking of patient status beyond conventional healthcare settings, like hospitals. The integration of blockchain in medical health facilitates the collection and sharing of data among healthcare stakeholders, ensuring transparency and accessibility of information [24,25].

Chain of Pharmaceutical Supply

Blockchain is applied in health care for managing drug or pharmaceutical supply chains, ensuring the safety and legitimacy of medical products introduced to the market and sold to end customers [26].

Digital Health Services

Blockchain contributes to the development of more secure, efficient, and patient-centric digital health services such as the Ayushman Bharat Digital Mission (ABDM), Aarogya Setu, and DigiLocker (Table 1).

**Table 1:** Digital health services in India.

S. No.	Name of program	Year	Launched under ministry	Aim	Objective
1	National Digital Health Mission (NDHM)	2020	Ministry of Health & Family Welfare	To establish a digital healthcare system in India, ensuring universal coverage, improved access, patient empowerment, and healthcare efficiency.	Creating a digital health ecosystem for improved healthcare access and quality. Empowering individuals with control over their health data. Promoting efficiency and transparency in healthcare services.
2	Ayushman Bharat Digital Mission (ABDM)	2021	Ministry of Health & Family Welfare	To digitize health care in India, improving accessibility and efficiency while empowering patients with digital health records.	To digitize health care in India, enhancing accessibility, interoperability, and efficiency, while empowering patients with digital health records.
3	Arogya Setu	2020	Ministry of Electronic & Information Technology	Help to prevent the spread of COVID-19 by providing users with important information, self-assessment tools, and alerts regarding potential exposure to the virus.	To serve as a digital contact tracing tool to identify potential COVID-19 exposure, provide self-assessment features, and disseminate relevant health information to users.
4	CoWIN App	2021	Ministry of Health & Family Welfare	The CoWIN app is to streamline the distribution and administration of COVID-19 vaccines in India.	To manage COVID-19 vaccine distribution effectively in India.
5	e-Sanjeevani	2021	Ministry of Health & Family Welfare	To offer remote telemedicine services, enhancing healthcare accessibility.	To enable remote healthcare consultations, improving accessibility and reducing the need for in-person visits.
6	e-Hospital	2015	Ministry of Electronic & Information Technology	To digitize and streamline hospital operations, including patient management, appointment scheduling, and medical records management, to improve efficiency and patient care.	To digitize hospital operations for improved efficiency and patient care.
7	Electronic Health Record	2013	Ministry of Health & Family Welfare	To digitize and centralize patient health information, making it accessible to healthcare providers for efficient and coordinated care delivery.	To improve healthcare quality and patient safety by facilitating accurate and efficient access to comprehensive patient information for healthcare providers.

Public Health Technology in Telehealth and Telemedicine

Blockchain enhances telehealth and telemedicine by providing decentralized, tamper-proof, transparent, and secure remote healthcare services. It ensures reliability, traceability, and trust in verifying healthcare professionals' credentials and the authenticity of home-based diagnostic testing kits, effectively preventing fraud [27].

Manage Financial Reports in the Hospital

Maintaining precise financial records is essential in bookkeeping, while efficient operation and evaluation of clinical trials are paramount. Blockchain firms have devised techniques to streamline accounting and reporting, offering a solution to the cumbersome paperwork process in health care. This innovation enables individuals to prepare documentation beforehand, potentially reducing waiting times at healthcare facilities. Yet, understanding the advantages and risks of blockchain's practical applications in health care is crucial for grasping its transformative impact on the system [27-29].

Use of Blockchain During COVID-19 and Public Health Emergencies

Blockchain's features, including decentralization and cryptographic security, enhance the handling of COVID-19 data, addressing challenges in providing accurate insights during the outbreak's moral crisis. Due to these concerns, the study highlights the importance of securing, maintaining integrity, and managing COVID-19 data in real time. This ensures patients receive the benefits denied to them due to misinformation [30]. Blockchain is also utilized for COVID-19 contact tracing. Patient information and health data are generated before and after various phases of clinical studies. Blood tests, quality assessments, estimations, and wellness surveys conducted on numerous individuals can yield results indicating the existence of certain documents or records. Healthcare providers review stored data and question its validity, seamlessly verifying it by comparing it to the original records stored on the blockchain system. Blockchain utilizes established cryptographic techniques to provide a suitable framework for secure data sharing. Healthcare providers record patient details such as name, date of birth, diagnosis, treatments, and ambulatory history in EHR format. This information is typically stored in cloud computing or existing databases [31-33].

Trust Immutability and Decentralization

Blockchain is inherently created to be transparent, decentralized, and unchangeable [34], and can enhance increased trust among stakeholders [35]. This holds in the situations outlined earlier. Through the removal of centralized decision-making, automation of processes, and enhanced transparency in the collection and utilization of data, blockchain has the potential to bolster trust in healthcare procedures [15].

Real-Time Disease Surveillance in Public Health

Blockchain enables real-time data sharing among healthcare providers, public health agencies, and researchers, facilitating timely detection and response to public health threats. We can stop widespread outbreaks and respond immediately to emerging health risks. Real-time disease surveillance in public health is a critical component of proactive healthcare management. Through the use of advanced technologies and systems like blockchain, we can enhance the timeliness and accuracy of disease surveillance efforts, ultimately safeguarding the health and well-being of communities worldwide. Real-time surveillance infrastructure and interdisciplinary collaboration will be essential to further strengthen our ability to detect, monitor, and mitigate the impact of infectious diseases on public health.

Health Insurance Claims

Health insurance is vital for cost-effective medical care, and blockchain's attributes, including immutability, decentralization, transparency, and audibility, can improve insurance claims; however, only 5% of the 22 chosen primary studies focused on this area. Zhou et al. introduced a blockchain-based health insurance storage system using Ethereum. This system encrypts and securely stores patients' insurance data on the blockchain, boosting credibility and removing third-party involvement in managing health insurance [26].

Limitations, challenges, and future scope of blockchain in public management

The primary obstacle to disseminating EHR for patient-centric research, market analysis, pharmaceutical investigations, healthcare data mining, and similar purposes lies in the concern for data privacy. There are several challenges in the application of blockchain technology in public health surveillance. These include data protection, future adaptability, legal concerns, and the requirement for strong guidelines on the proper utilization of blockchain solutions.

Researchers have grappled with the challenge of managing extensive datasets and safeguarding patient privacy for an extended period of time. In contrast, blockchain technology has mitigated certain issues by offering a secure and decentralized platform. Regrettably, current EHR management systems face challenges related to data manipulation, delayed communication, and a lack of trust in collaborative efforts for data collection, storage, and distribution. The issues surrounding healthcare data privacy are discussed in this section, along with an analysis of the laws that are already in effect and those that will be implemented shortly. It also gives a summary of the architecture, present issues, and future uses of blockchain technology for managing and protecting the privacy of medical health records that are created now and in the future. The chapter goes on to present several blockchain solutions that support future directions for big data, blockchain integration, and healthcare research [36]. Understanding the inherent properties of blockchains is crucial, but equally important is addressing domain-specific challenges for practical applications. This section explores four key interoperability challenges in blockchain-based healthcare apps: system evolvability, blockchain data storage, healthcare information privacy, and system scalability [37]. The integration of blockchain technology into the healthcare sector presents certain challenges that need to be overcome. One major obstacle to the adoption of this advanced technology by medical facilities is the shortage of expertise. Blockchain applications are still in their infancy and require further exploration and research. This applies particularly to the responsibilities of medical associations and regulators. The healthcare sector must advance. The future expansion of blockchain in health care is highly probable. As this technological innovation evolves, its applications in health care will enhance outcomes and progress in the treatment process. Blockchain technology plays a central role in validating transactions and information transfers [23].

In the coming days, blockchain technologies will allow transactions to be verified and recorded with the approval of network members. This technology will ensure strong security through public and private key encryption, safeguarding patient information at a new level for sharing health data. Blockchain technology has the potential to improve public health management, making health care better, safer, and more accessible for everyone. Blockchain holds promise for improving various aspects of health care, including managing patient records, preventing breaches, enhancing interoperability, streamlining processes, overseeing medications and prescriptions, and monitoring medical and supply chains. The potential of blockchain in health care is expected to yield significant advancements in the future (Table 2) [23].

**Table 2:** List of included studies in the review.

S. No.	Author	Year	Type of article	Findings
1	Ghorashi et al. [2]	2023	Original article	The study highlights a knowledge gap in radiology, and an interest in blockchain and smart contracts, and suggests addressing the gap for improved security and patient care.
2	Benchoufi et al. [20]	2017	Original article	Using blockchain technology for consent collection streamlines the process by creating an unforgeable mark on the blockchain. This enhances data accuracy and transparency, giving patients more control over their consent.
3	Dar et al. [30]	2022	Original article	Healthcare data system prioritizes patient privacy with blockchain, using cryptographic functions for anonymity and protection of data.
4	Tripathi et al. [10]	2020	Original article	Blockchain and other advanced tech can transform health care into a secure, decentralized system, improving services.
5	Cunningham et al. [17]	2017	Ebook	Identified requirements for patient-controlled electronic health records led to an application programming interface prototype using distributed ledger technology. Despite challenges, like inefficiency, it offers unique trust and security solutions in health informatics, with growing adoption ahead.
6	Dauda et al. [1]	2021	Review article	Blockchain in health care improves data security, autonomy, and exchange. It enables electronic health records and enhances medical treatment assessment. Despite challenges, acceptance and integration are increasing.
7	Elangovan et al. [16]	2022	Review article	Blockchain is beneficial in health care, supporting electronic records, research, patient tracking, and supply chains, addressing data security needs.
8	Velmovitsky et al. [15]	2021	Review article	Blockchain addresses healthcare challenges through data immutability and efficiency, optimizing processes, reducing inefficiencies, and enhancing trust.
9	Haleem et al. [23]	2021	Review article	Blockchain in health care secures records, improves interoperability, combats counterfeit drugs, streamlines insurance mediation, and accelerates clinical actions.
10	Chang et al. [35]	2020	Review article	Blockchain in health care enables stakeholder coevolution, value creation, and sharing, requiring further empirical research for validation.
11	Liang et al. [24]	2017	Complete book	Paper introducing mobile healthcare system with blockchain for secure data handling and Hyperledger Fabric for communication. Future integrates personal/medical data.
12	Liu et al. [18]	2018	Complete book	In this study, blockchain-based privacy-preserving data use blockchain for secure electronic medical records sharing with privacy, storing records in a tamper-proof blockchain for security and privacy.
13	Barrett et al. [7]	2013	Review article	Big data in disease prevention uncovers personalized risk factors, promotes behavior change, enhances public health, and lowers healthcare costs. Urgency arises from an aging population, rising costs, and chronic illness impact.
14	Ahmad et al. [27]	2021	Review article	Early stages of blockchain in telehealth/telemedicine pose obstacles. Research is needed to facilitate the widespread use of blockchain in these systems.
15	Al Omar et al. [29]	2017	Book chapter	A healthcare data management system that prioritizes patient focus, utilizes blockchain for privacy, and ensures anonymity through cryptographic functions.

## Conclusions

Blockchain technology is essential for decentralization, and encryption facilitates new applications in the healthcare sector. It increases connectivity between healthcare organizations, improves the economic value of health information, and enhances the security of patient's computerized medical data. Blockchain technology has the potential to enhance patient history management, particularly in the areas of monitoring, electronic health records, biomedical research, education, insurance claims, pharmaceutical supply chains, patient identification, data handling, and remote patient monitoring, which will speed up healthcare processes while maintaining the most effective data quality.

The summary shows that blockchain can make health records safer, more private, connected, and more efficient. The application of blockchain has provided long-term benefits through improvements and interactions with other emerging technologies. Blockchain protects electronic health records while empowering people to control their health data, monitor patients remotely, and maintain healthcare transparency. Public health could see significant changes as a result of blockchain technology. In general, blockchain has the potential to completely transform public health surveillance, resulting in improved health outcomes for communities globally. Blockchain technology deals with concerns about patient empowerment, secure communication, efficiency in processes, and data security.

## Figures and Tables

**Figure 3:**
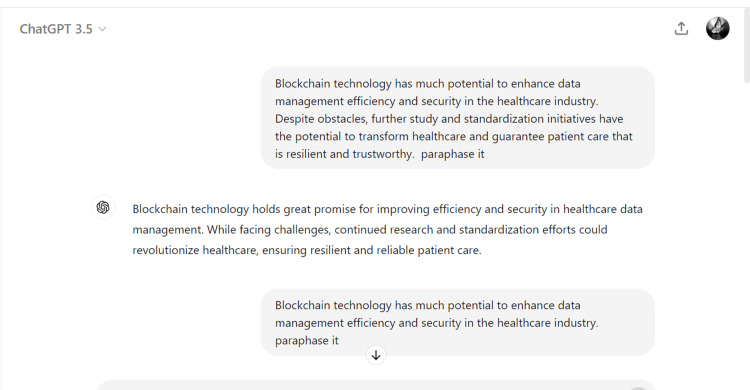
Screenshot of artificial intelligence use (ChatGPT) for paraphrasing.
